# The Durability of Cognitive Behavioral Therapy for Insomnia in Patients with Chronic Pain

**DOI:** 10.1155/2012/679648

**Published:** 2012-08-09

**Authors:** Carla R. Jungquist, Yolande Tra, Michael T. Smith, Wilfred R. Pigeon, Sara Matteson-Rusby, Yinglin Xia, Michael L. Perlis

**Affiliations:** ^1^School of Nursing, University at Buffalo, Wende Hall 304, Buffalo, NY 14214, USA; ^2^Sleep & Neurophysiology Research Lab, University of Rochester Medical Center, Rochester, NY 14642, USA; ^3^Maryland Poison Center, School of Pharmacy, University of Maryland Baltimore, Baltimore, MD 21201, USA; ^4^Department of Psychiatry and Behavioral Sciences, John Hopkins University, Baltimore, MD 21287, USA; ^5^VA Center of Excellence for Suicide Prevention, Washington, DC 20420, USA; ^6^Department of Biostatistics and Computational Biology, University of Rochester School of Medicine, Rochester, NY 14642, USA; ^7^Department of Psychiatry, University of Pennsylvania, Philadelphia, PA 19104, USA

## Abstract

The purpose of this study was to assess the long-term (six months) effects of cognitive behavioral therapy for insomnia (CBT-I) in patients with chronic pain. The results of the pre-post treatment effects have been reported previously. The therapy was delivered by an advanced practice nurse in a research setting using a parallel-group, randomized, single blind trial of CBT-I with a contact/measurement control condition. Outcomes included sleep diary, the Insomnia Severity Index, the Multidimensional Pain Inventory, the Beck Depression Inventory, the Profile of Mood States-short form, and the Pain Disability Index. Measurement time points were end-of-treatment, three-month and six-month posttherapy. Subjects receiving CBT-I (*n* = 19), as compared to control subjects (*n* = 9), did not exhibit any significant group by visit effects on measures of sleep, pain, mood, or function after end of treatment. However, subjects in the treatment group exhibited statistically (*P* = 0.03) and clinically significant improvement in total sleep time (23 minutes) over the six months following treatment. In this paper, cognitive behavioral therapy directed to improve insomnia was successfully delivered to patients with moderate-to-severe chronic pain and the positive effects of CBT-I continued to improve despite the presence of continued moderate-to-severe pain.

## 1. Introduction

Chronic Pain is a common condition that affects the quality of life of more than 76.2 million Americans [1]. Treatments are often palliative in nature and are focused on improving function and quality of life as opposed to curative in nature. Sleep, known to be important to achieve optimal quality of life, is inevitably disturbed in the presence of pain. Approximately 53% of patients who live with chronic pain also experience sleep disturbance [2–5]. Traditionally, clinicians have indirectly addressed sleep disturbance in the presence of pain by treating the painful condition. In fact, the effectiveness of treatments for pain is often judged on a patients' improvement in sleep. This practice seems logical, as nociceptive arousal is a precipitator and potentially a perpetuator of insomnia. But the concern is that patients may become overmedicated by the practice of titrating pain medications according to improvements in sleep as well as pain. As a clinician using cognitive behavioral interventions for insomnia (CBT-I), there is also the concern that sleep restriction may potentiate pain, or that the gains, if any, achieved from CBT-I would be short lived due to the continued nociceptive stimulation from the painful condition. Currently, there is little evidence to base clinical practice guidelines on whether to treat sleep disturbance comorbid with pain using pain treatments, or treat sleep disturbance directly. 

To begin to address this lack of evidence, there have been a few clinical trials conducted to assess the efficacy and safety of cognitive behavioral therapy for insomnia in patients with chronic pain. These studies also assessed if improvements in sleep would also be associated with improvements in pain. The findings are mixed and draw on a relatively small number of subjects. A secondary analysis by Vitiello and colleagues of cognitive behavioral therapy for insomnia reported that CBT-I improved sleep complaints, and some, but not all measures of pain severity in patients with osteoarthritis [6]. Whereas a study of heterogeneous pain patients and a clinical trial of fibromyalgia both showed significant improvements in sleep complaints, but failed to demonstrate that CBT-I improved pain [7–9]. 

We conducted a clinical trial of CBT-I in chronic back and neck pain patients comparing subjects receiving CBT-I (*n* = 19), to control subjects (*n* = 9). Results of the baseline to posttreatment effects were published previously [10]. To summarize those findings, baseline to posttreatment between group comparisons showed that subjects in the treatment group exhibited significant decreases in sleep latency, number of minutes awake after sleep onset, number of awakenings, sleep quality as measured by the insomnia severity index, and a significant increase in sleep efficiency and daytime vigor. Total sleep time was not significantly altered. Despite continued moderate-to-severe pain, the observed effect sizes for the sleep outcomes were comparable to or better than meta-analytic norms for subjects with primary insomnia. We, however, did not discern significant short-term effects on pain severity, although there were moderate positive treatment effects. In this report, we analyze the three and six-month follow-up data from this trial to assess if the presence of pain interfered with the long-term effects of CBT-I. We also assessed the duration of effects CBT-I had on sleep, mood, function, and pain and analyzed possible interrelationships among those variables over time using multilevel and statistical mediation models to ascertain whether longer-term improvements in pain might be mediated by changes in sleep and/or mood. 

## 2. Materials and Methods

This study was approved by the Research Subjects Review Board at the University of Rochester and all subjects underwent the informed consent process. Data was collected from 2003–2005.

### 2.1. Design

Patients, age 25 or older, with insomnia comorbid with nonmalignant chronic (>6 months) pain were recruited from the community and local pain treatment clinics to participate in a parallel-group, randomized, single blind trial of cognitive behavioral therapy for insomnia (CBT-I) with a contact/symptom monitoring and discussion control condition. Exclusion criteria included presence of AHI > 10 or presence of other intrinsic sleep disorders on baseline PSG. 

*Treatment* (*CBT-I*) consisted of 8 sessions of CBT-I including sleep restriction, stimulus control, cognitive therapy, and sleep hygiene and was delivered individually by an advanced practice nurse in a research setting. Treatment procedures were previously reported [10].

*Control condition* consisted of 8 sessions to serve the purpose of control for the effects of therapist contact and filling out a sleep/pain diary, and to allow the therapist to monitor patient safety (suicidal thoughts/worsening depression). During each session, subjects reviewed their sleep/pain diary data, and their Beck Depression Inventory items that were scored above 0 for the prior week with the therapist. Sessions lasted between 30–90 minutes and time with the therapist was the same regardless of group assignment. The first two sessions lasted about 90 minutes and each following session was 30 minutes. The review was interrogative in nature and did not prescribe or suggest behavior change. An example is as follows, “*this week I see that your average pain severity score was higher than last week; what factors do you think contributed to this?*” In order to control for expectancy, subjects in the contact control arm of the study were told that by attending to events which correspond to pain and/or mood change, their capacity to be insomnogenic or depressogenic (kindle new episodes of insomnia and depression) would be reduced. Integrity and treatment fidelity was monitored by a consultant who reviewed video tapes of a random sample of sessions.

### 2.2. Measures

Sleep diaries, actigraphy, and other questionnaires were administered at intake, end of treatment, and at three and six months posttreatment. Patients recorded their sleep variables daily on diaries for 2 weeks pretreatment through 2 weeks posttreatment, then for 2 weeks at 3 and 6 month posttreatment end. Sleep diary variables included time to bed, time out of bed, minutes to fall to sleep (SL), minutes awake after sleep onset (WASO), and number of awakenings (NWAKs). Total sleep time (TST) and sleep efficiency (SE) were calculated by the therapist.

*Actigraphy* was used to obtain an objective measure of sleep continuity to corroborate with subjective report of sleep continuity. Mini-Mitter score actiwatch were worn on the nondominate wrist 24 hours a day over the same timecourse as sleep diary acquisition. Subjects were asked to press the mark button at time to bed and time out of bed. Epochs were recorded every minute and Mini Mitter software was used to calculate sleep and daytime motion (average activity counts per minute) that was used for analysis. 

*Insomnia severity index (ISI)* is a 7-item subjective measure of sleep disruption, satisfaction, and worry about sleep and how sleep interferes with daytime function. Each item is rated on a 0–4 scale with 0 representing no symptom and 4 representing very much. The threshold for mild insomnia is a score ≥7 [11]. A change in score of 7 points is thought to be clinically relevant [12].

*Multidimensional pain inventory (MPI)* is a 60-item self-report inventory designed to assesscognitive, behavioral, and affective response topain. The MPI consists of 12 scales. Two of the scales (pain severity and pain interference) were used in the present analysis. The pain severity scale consists of three questions about pain intensity which are scored 0 = none to 6 = very much. The sum score is divided by the number of questions to obtain the average scale score. An example of a question from this scale is “Rate the level of your pain at the *present moment.*” The mean norm for this scale in chronic pain patients is 4.2 ± 1.2 [13, 14]. The interference scale assesses how pain interferes with activities of daily living, work, life, and social functioning. The scale consists of 9 questions each of which are scored on a 0 to 6 scale. The sum score is divided by the number of questions to obtain the average scale score. An example of a question from this scale is “In general, how much does your pain interfere with your day-to-day activities.” The mean norm for this scale in chronic pain patients is 4.0 ± 1.5. A change in score of 1 point is considered clinically relevant [15].

*Pain disability index (PDI)* is a 7-question instrument that measures the degree that pain interferes with functioning. It is scored on a 0–10 numeric scale where zero means no disability at all, and a score of 10 signifies that all of normal activities have been totally disrupted or prevented by pain [16, 17]. Total score ranges 0–70.

*Profile of mood states (POMS)* short form is a 30-item measure of affective states for the past week. The subjects rated their symptoms on a scale of 0 (no at all) to 5 (extremely). The instrument consists of six subscales of 5 items each. The subscales total possible score is 20. This instrument has been used in numerous studies and has been found to be test-retest reliable and have concurrent validity with predictive construct [18]. Two subscales representing vigor and anxiety were used in this analysis. The vigor items include: full of pep, vigorous, energetic, active, and lively. The anxiety items include: tense, shaky, uneasy, nervous, and anxious. 

*Beck depression scale II (BDI)* is a 21-item measure of depression symptoms. The range of score is 0–63 with a clinically relevant threshold for depression ≥15 [19, 20]. 

*Determination of treatment response* parameters for treatment response per sleep diary variables were determined using outcomes from previously published studies of CBT-I [21]. Parameters for insomnia severity index were determined according to published guidelines [22].

## 3. Data Analysis

Using logistic regression analysis of group differences at time point post treatment, it was determined that gender and age and were significantly different due to attrition during treatment. Therefore, covariates used in the analyses included age gender, in addition to change in medication over the course of the study and change in medical or pain problems over the course of the study. Age was reported in years and determined at the day of initial study visit. To address the possible confounding effect of subjects' changing their medications or developing worsening or resolution of their chronic pain, medication use change and change in medical/pain condition was tracked at each visit using a yes/no question. Questionnaires tracking all medications with their doses as well as medical conditions were tracked at baseline, 3 month, and 6-month visits. Variables were developed to represent change in medication between intake and 3-month follow-up visit; change in medication between three- and six-month follow-up visits; change in medical problems between intake and three-month visit; and change in medical problems between three-month and six-month visits. Variable was coded yes or no. 

All the data analyses were conducted using SAS 9.2 version (SAS Institute Inc, Cary, NC, USA). Logistic Regression was performed to determine if characteristics of the subjects would predict group assignment. Age, gender, medication change between baseline and 3 month, and between 3 month and 6 month were initially entered in the model as covariates, and backward elimination procedure was used to derive the final model. In order to reduce selection bias, any covariate with *P*-value < 0.10 was kept in the final models.

There are two well-established missing data mechanisms to detect the impact of missing data: missing completely at random assumption (MCAR) and missing at random assumption (MAR). Generalized estimating equations (GEEs) approach was used to perform the analyses. Since the validity of GEE estimates depends on whether MCAR is met, before applying the GEE approach to the data the MCAR assumption was tested against the more general MAR by modeling the missingness of the patients' response as a function of observed responses using logistic regression. If the results of logistic model show that missingness depends on the observed responses at baseline, then MCAR is deemed inappropriate and the weighted generalized estimating equations (WGEEs) was used instead, with weights estimated from the logistic model for missing data [23]. In the present study, there was no evidence to reject MCAR and, therefore, the GEE procedure was used to assess duration of treatment effects for each of the outcomes. The posttreatment to 6-month effects were modeled using the group-by-time interaction term using PROC GENMOD in SAS. Score statistics for Type 3 GEE analysis evaluated significance of group effects during followup visits. If there was evidence of an effect, the differences of least squares means were used to compare and describe the effect. Additionally, mediation models were run on the sample as a whole as pain reduced over time in both groups (no group effect). The method used the ***mlm*** option in Mplus that provided a mean-adjusted chi-square model test statistic. All time points (baseline, post treatment, 3-month and 6-month followup) were included in this mediation analysis.

## 4. Results

**Figure 1:**
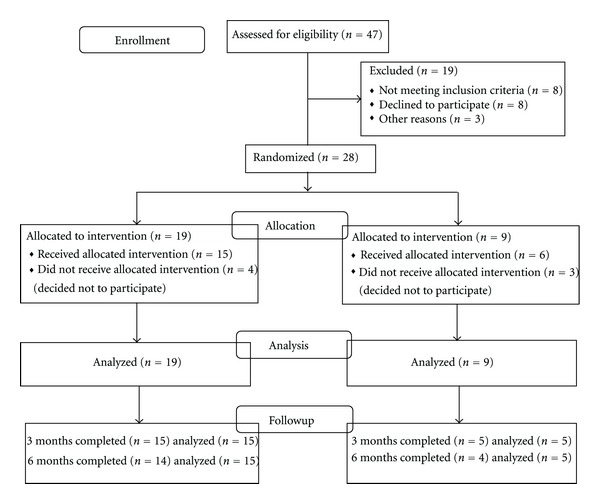
Subject flow diagram.

Forty-seven (*n* = 47) subjects with chronic nonmalignant pain located in the spinal region (neck and back) were consented during the screening phase of the study. Twenty-eight subjects met study eligibility criteria and were randomized (~2 : 1) to either CBT-I or a contact/measurement control condition (19 CBT-I, 9 controls). See Figure 1. Study eligible subjects were double match randomized by a blinded third party until the 16th subject was reached; at that point a stratification procedure according to gender, age, and ethnicity occurred. The double-matching procedure was used so that a larger sample of subjects could be exposed to the experimental condition.

See Table 1 for sample characteristics. In-depth discussion of sample including medication use was previously published [10].

*Change in medication use or medical problems over the course of the study.* All subjects were taking pain medications. Although subjects were strongly encouraged to maintain their medications patterns over the course of the study, five subjects changed their pain medications between intake and the three-month visit. Of those five subjects, two decreased their pain medications, two increased their pain medications, and one person changed the type of pain medication being taken. Three subjects changed their pain medications between the three- and six-month follow-up visits. Of the three subjects, two decreased their medications and one increased their medication. At three months, two subjects reported a change in medical condition. At the six-month visit, one subject reported a change in medical condition. Medication change was included as a covariate in the GEE analysis.

### 4.1. Outcomes on Measures of Sleep 

*Corroboration of subjective and objective measures of sleep variables. *Using *t*-test analysis and results of GEE analysis, average weekly over the course of the study subjective sleep measures as reported on sleep diary were not found to be significantly different from objective measures (actigraphic) on variables of total sleep time and sleep latency. However, there were significant group-by-time differences (*P* < .05) on measures of wake after sleep onset and sleep efficiency at time points posttreatment and 3-month followup. See Table 2 for means (sd). Actigraphy consistently sensed the subjects were awake more than subjective diary report. 

**Figure 2:**
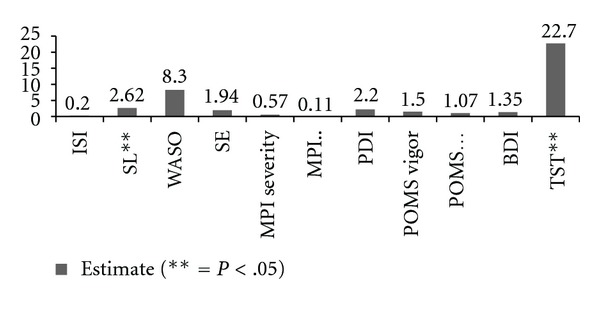
Change score end of treatment to six-month followup.

*Posttreatment to end of study changes per GEE analysis.* See Table 3 for group means (controlling for covariates) and Figure 2 for change scores estimate (from end of treatment to 6-month followup). As per Figure 2, subjects in the treatment group continued to increase their total sleep time by an additional 23 minutes (controlling for all covariates) despite continued moderate-to-severe pain. Significant differences from posttreatment to end of study were seen on sleep latency and total sleep time only. 

*Insomnia status posttreatment and end of study. *Most of the subject's insomnia remained remitted at the 6-month follow visit. See Table 4 for responder status per sleep diary and insomnia severity index over the course of the study. As there is no one definition of remission, we have included remission status using thresholds on the insomnia severity index, comparing it to thresholds on sleep diary two-week averages at each time point. 

*Outcomes on measures of pain. *The multidimensional pain inventory (MPI) subscores for pain severity and interference secondary to pain and the total score on the pain disability index were used for analysis. No significant group-by-visit effects were seen after end of treatment. 

*Outcomes on measures of mood. *Mood was measured by the Profile of Moods State (anxiety and vigor subscales) and the Beck Depression Inventory total score. No significant group-by-visit effects were seen after end of treatment on the measures of depression and anxiety. 

### 4.2. Post-Hoc Analysis

*Post-Hoc mediation model analysis. *Mediation models were run on the sample as a whole as pain reduced over time in both groups (no group-by-time effect). It was of interest to know if depression mediates the effect of the treatment (CBT-I) on pain severity. An autoregressive mediation model was considered: the CBT-I intervention program (Group) was the independent variable (X), depression (BDI total score) was the mediating variable (M), MPI pain severity was the dependent variable (Y) 12, 13. Longitudinal mediation across time period used the temporal precedence of X to M to Y. Since Group is categorical, the standardized estimate of Y is reported. Indirect effects were tested with bootstrapping. The analyses were conducted using the Mplus 5.2 [24]. All time points (baseline, 8-weeks, 3-months and 6-months followup) were included in the mediation analysis. *Results. *Among all longitudinal mediations, there was a significant indirect (mediated) effect (*P* = 0.025, with 95% lower and upper confidence limits of 0.055 and 0.827) of the intervention on pain at 3 months through depression at 8 weeks. The pain at 3 months for control group is 0.441 standard deviations units higher than treatment group through the effect of depression at 8 weeks. This implies that changes in pain severity at 3 months were moderated by improvement in depression. 

*Post-Hoc analysis of treatment group activity. *During the course of the study, subjects in the treatment group consistently reported to the therapist that they felt so much better that they were more active, resulting in continued or at times increased pain. We explored the relationship between pretreatment daytime activity as measured by actigraphy (wake activity counts) and activity at 6-month followup in the treatment group. Activity count during wake at pretreatment [M 61 (sd 19)] compared to six-month followup [M 82 (sd 43)] trended towards significance via paired *t*-test analysis [*t* = −2.03, df 12, *P* = .06].

## 5. Discussion

This study reported the posttreatment findings of our previously published study. This report provides evidence that the positive sleep effects of CBT-I in patients with chronic pain are sustained for at least six months despite continued moderate-to-severe chronic pain. These findings strengthen the argument that insomnia co-morbid with chronic pain is exactly that: comorbid as opposed to secondary. As per the Spielman Model, pain and the stressors that go along with life style changes from living with chronic pain are likely precipitators for the development of insomnia, but once developed, insomnia takes on a life of its own. When treated with CBT-I, insomnia resolves and total sleep time continues to improve over time despite the presence of continued pain. These findings of the duration of positive effects on sleep are consistent with several other studies of CBT-I in patients with primary insomnia as well as in insomnia comorbid with cancer, addiction, depression, PTSD, and other pain conditions [6, 25–33]. The relationship between improvements in sleep and their direct effect on subjective ratings of chronic pain continues to be less than convincing. As published previously, in our original analysis of the pre-post treatment response, we found significant group-by-time differences in measures of function related to pain (MPI interference scale). Lack of group-by-time findings in our follow-up data on measures of pain may be due to a number of factors such as the complexity of measuring pain, the multidimensional aspects of pain, the relatively poor response of pain to a variety of pain treatments, or that treatment of insomnia has no direct effect of the perception of pain severity. Perhaps the small effects on pain that were seen pre-post and have been found in other studies are in-direct effects through improvements in general quality of life, physical function and energy, and/or improvements in depressive symptoms [6, 9, 34]. In a preliminary attempt to explore relationships of pain, sleep, and measures of mood and activity, we performed a mediation analysis. Recognizing that a weakness of the analysis is our sample size, the findings support the theory that the marginal improvements in pain may have been through improvements in depression [35]. This is not a surprising finding, as it is well known that mood and pain have a strong and positive relationship. Improvements in mood and pain seen in our control group lead us to suspect that our control condition may have provided active treatment for psychological factors including an effect on pain. There is evidence that written or verbal expression of emotions has produced benefits on pain in other studies [36–38]. Additionally, efficacy studies of CBT-I have found improvements in depressive symptoms [28, 29]. 

Further research in the relationship between sleep and pain is needed to understand the underlying pathophysiology and how it relates to often highly correlated symptoms of depression, fatigue, energy, anxiety, and physical/emotional function. Developing further understanding of the relationships will allow for the development of interventions that may supplement the treatment effects of our standardized CBT-I approach. Additionally, the use of standardized measures and reporting of sleep continuity, pain, and depression variables throughout all studies would be helpful to this line of research. Actigraphy continues to be a valuable tool but discrepancies between subjective report and objective actigraphic measures confound the analysis. In this study, we found actigraphy to be helpful in visualizing the subjective circadian rhythm pattern as well as sleep continuity variables to assure compliance with treatment procedures. The discrepancies between subjectively reported and objectively recorded WASO and SE in this study could be the result of limb movements often associated with chronic pain of the neck and back. The differences between sleep diary and actigraphy seen in this study are not consistent with other studies in subjects with primary insomnia and/or fibromyalgia [9, 26]. Actigraphy was also used in this study to determine if the treatment group was on average more active posttreatment than pretreatment. Although a marginally significant difference was found, with activity increasing pre- to posttreatment, the clinical relevance is unknown. Further research is needed to determine reasons for discrepancies in various populations, as well as clinically relevant changes in daytime activity count. This study was affected by difficulty with recruitment and subsequent attrition, and thus a small sample size. Notably, there was a higher percentage of subjects from the control condition that withdrew after treatment started as compared to the CBT-I condition. At time of withdrawal, most subjects reported “no time” to participate in the study. Research using behavioral interventions can be difficult for some subjects in general due to the number of study visits and behavioral sleep intervention studies typically further burden subjects with the need for daily recording of sleep variables. The attrition from this study underscores the need to design studies and interventions that are less time consuming for the therapist and the subject. Although it could be that attrition would not be as much of a problem in clinical practice, where patients are seeking service and paying for it. Pioneering investigators have performed a preliminary study introducing brief forms of treatment delivery [26, 39, 40]. Now that there is sufficient evidence that CBT-I is helpful for patients with chronic pain, studies of its application in the clinical setting are needed. As the relationship between sleep disturbance, depression and pain severity is complex, it would be helpful to consider addressing this relationship for future interventional studies. 

This study provides evidence that CBT-I can be delivered effectively to patients with chronic pain, and the effects continue to improve in the long-term despite continued pain. Future research is needed on the application of therapy in the clinical setting. The multidisciplinary pain clinic setting naturally lends itself to the introduction of CBT-I as a possible stand-alone or adjuvant intervention for pain patients. This can be accomplished by pain psychologists or, as this study supports, by advanced practice nurses. Adding CBT-I to the skill sets of behavioral providers in pain clinics will likely improve patient outcomes.

## Figures and Tables

**Table 1:** Sample characteristics.

	Posttreatment		3-month followup		6-month followup	
	Control *N* = 6	Treatment *n* = 15	Control *n* = 5	Treatment *n* = 15	Control *N* = 4	Treatment *n* = 14
Age (years)	41 (11)	52 (9)	41 (12)	52 (9)	42 (14)	52 (10)
Gender (female)	83%	73%	80%	73%	75%	71%
BMI	27 (6)	28 (3)	28 (6)	28 (3)	26 (4)	28 (4)
Occupation						
Disabled	2	2	1	2	1	2
Work FT	3	8	3	8	2	7
Work PT	1	3	1	3	1	3
Retired	0	2	0	2	0	2
Race/Ethnicity						
AA	2	1	1	1	1	1
Caucasian	4	14	4	14	3	13
Hispanic	0	0	0	0	0	0
Education						
HS	2	3	1	3	1	3
AA	2	7	2	7	1	6
BS	1	4	1	4	1	4
MS/PhD	1	1	1	1	1	1

**Table 2:** Comparison of sleep diary and actigraphy: two-week mean (sd).

	SL		WASO		TST		SE	
	Sleep diary	Actigraph	Sleep diary	Actigraph	Sleep diary	Actigraph	Sleep diary	Actigraph
Post tx *n* = 12	15 (13)	18 (21)	19 (18)^∗^	53 (21)^∗^	406 (52)	370 (59)	90 (9)^∗^	79 (10)^∗^
3 mo. *n* = 20	18 (17)	19 (14)	23 (27)^∗^	57 (29)^∗^	412 (69)	379 (62)	88 (12)^∗^	79 (9)^∗^
6 mo. *n* = 16	17 (19)	16 (8)	32 (55)	56 (20)	413 (65)	387 (37)	88 (13)	81 (5)

SL: sleep latency; WASO: wake after sleep onset; TST: total sleep time; SE: sleep efficiency ^∗^(*P* < .05).

**Table 3:** Least-square Means (sd) by group.

Variable	Posttreatment		3 Months		6 Months	
	Treatment *n* = 19	Control *n* = 9	Treatment *n* = 15	Control *n* = 5	Treatment *n* = 14	Control *n* = 4
SL	9 (1)	46 (11)	14 (2)	31 (10)	12 (1)	42 (15)
WASO	11 (2)	42 (1)	17 (3)	41 (18)	19 (4)	78 (53)
SE	94 (1)	73 (5)	91 (1)	78 (7)	92 (1)	76 (10)
TST	408 (3)	344 (31)	433 (13)	349 (28)	431 (9)	344 (47)
ISI	3 (1)	11 (2)	3 (1)	12 (1)	3 (1)	9 (1)
MPI Severity	2.4 (.4)	3.8 (.5)	2.6 (.3)	3.8 (.6)	3.0 (.4)	3.7 (.3)
MPI Interference	2.9 (.3)	3.4 (.7)	2.8 (.2)	2.8 (.8)	2.8 (.2)	2.4 (.8)
PDI	19 (5)	35 (4)	20 (4)	28 (7)	21 (4)	25 (5)
POMS Anxiety	1 (.4)	9 (7)	1 (.4)	2 (1)	2 (.6)	0 (.4)
POMS Vigor	11 (1)	7 (2)	13 (1)	7 (2)	13 (1)	6 (1)
BDI	2 (.8)	5 (2)	3 (.6)	8 (2)	3 (1)	8 (4)

**Table 4:** Response of subjects completing all visits.

	Posttreatment *n* = 15	End of study *n* = 14
*Sleep diary*		
Non responder	0	0
Treatment responder (43% SL; 55% WASO improvement)	5	1
Remitter (SL and WASO ≤15)	10	10
Relapse (>30 SL or WASO)	Not applicable	3

*Insomnia severity index*		
Nonresponder	1	1
Treatment responder (ISI change score >7)	1	0
Remitter (ISI < 7)	13	10
Relapse	Not applicable	3
